# Endovascular treatment for acute ischaemic stroke due to medium vessel occlusion: data from ANGEL-ACT registry

**DOI:** 10.1136/svn-2022-001561

**Published:** 2022-09-01

**Authors:** Dapeng Sun, Raynald ­, Xiaochuan Huo, Baixue Jia, Xu Tong, Anxin Wang, Gaoting Ma, Ning Ma, Feng Gao, Dapeng Mo, Zhongrong Miao

**Affiliations:** 1 Department of Interventional Neuroradiology, Beijing Tiantan Hospital, Capital Medical University, Beijing, China; 2 Department of Neurology, Beijing Tiantan Hospital, Capital Medical University, Beijing, China; 3 Department of Neurosurgery, Beijing Fengtai You anmen Hospital, Beijing, China; 4 China National Clinical Research Center for Neurological Diseases, Beijing Tiantan Hospital, Beijing, China

**Keywords:** Stroke, Thrombectomy

## Abstract

**Objectives:**

To investigate the safety and efficacy of endovascular treatment (EVT) for acute medium vessel occlusion (MeVO) in the anterior circulation and to explore the independent predictors of the 90-day good outcome for such patients.

**Methods:**

Data from ANGEL-ACT Registry were analysed in our study. The outcomes, such as the modified Rankin Scale (mRS) at 90 days, successful recanalisation rate and symptomatic intracranial haemorrhage (SICH) rate, were compared between MeVO and acute large vessel occlusions (LVO). Then, the independent predictors of the good outcome at 90 days in MeVO patients were determined by the logistic regression analyses.

**Results:**

We included 1032 subjects in the analysis, of which, 147 were MeVO and 885 were LVO. mRS at 90 days distribution (3 (0–4) vs 3 (0–5), common odds ratio (OR) =1.00, 95% confidence interval (CI) 0.73 to 1.38, p=0.994), SICH rate (4.8% vs 8.9%; OR=0.59, 95% CI 0.26 to 1.34, p=0.205) and successful recanalisation rate (89.8% vs 89.7%; OR=1.00 95% CI 0.51 to 1.93, p=0.992) were similar between the MeVO and LVO groups after adjusting for the confounders. We identified that baseline neutrophil-to-lymphocyte ratio ≤4.1 (OR=2.13, 95% CI 1.14 to 3.99, p=0.019), baseline National Institutes of Health Stroke Scale ≤14 (OR=1.96, 95% CI 1.02 to 3.80, p=0.045) and mechanical thrombectomy passes ≤1 (OR=2.16, 95% CI 1.14 to 4.11, p=0.021) were independent predictors of the 90-day good outcome in MeVO patients undergoing EVT.

**Conclusions:**

Patients with MeVO achieved similar 90-day mRS, SICH rate and successful recanalisation rate after EVT compared with patients with LVO. Several independent predictors of 90-day good outcome in MeVO patients undergoing EVT were determined, which should be highly considered in MeVO stroke management.

**What is already known on this topic**

- The efficacy and safety of endovascular treatment (EVT) for acute medium vessel occlusion (MeVO) are still unclear, even though several single-centre retrospective studies have been reported. Some predictors influencing the outcomes of MeVO patients receiving EVT, such as age, undergoing intravenous thrombolysis or not, and infarct volume, have been previously found.

**What this study adds**

- The current study based on a multicentre prospective database in China suggested no significant differences in 90-day modified Rankin Scale, symptomatic intracranial haemorrhage rate and successful recanalisation rate between MeVO and large vessel occlusion undergoing EVT. We added several new predictors influencing the outcomes of MeVO, such as baseline National Institutes of Health Stroke Scale, neutrophil-to-lymphocyte ratio and the number of mechanical thrombectomy passes.

**How this study might affect research, practice or policy**

- Our study identified some predictors of the good outcome at 90 days for MeVO, which should be highly considered during MeVO management. However, further large randomised controlled trials are warranted.

## Introduction

Several well-known randomised controlled trials (RCTs) have suggested the efficacy and safety of endovascular treatment (EVT) for anterior circulation acute proximal large vessel occlusion (LVO).^1^ However, there are some concerns regarding the benefit of EVT for other vessel occlusions beyond LVO, such as medium vessel occlusion (MeVO), due to exclusion or small sample size in these RCTs.^1^ MeVO is defined as the occlusions of the anterior cerebral artery (ACA) A2/A3 segments, middle cerebral artery (MCA) M2/M3 segments and posterior cerebral artery P2/P3 segments, accounting for 25%–40% of acute ischemic stroke (AIS).^2^ Although MeVO stroke has better functional outcomes than LVO stroke, about 33% of MeVO without EVT still cannot achieve a good outcome at 90 days; specifically, for proximal M2 occlusion, the 90-day good outcome only accounts for 50%.^3^ Moreover, the characteristics of medium vessels, such as distal location, lower diameter and thinner walls^4^ have made it a technical challenge. Nevertheless, using advanced neurointerventional techniques and devices, MeVO is now emerging as a promising next potential EVT goal.

The efficacy and safety of EVT for MeVO have been studied extensively, with most publications being retrospective and single-centre studies.^5–13^ Thus, their findings might not be sufficiently applicable to real-world practice. As a result, a multicentre study is required to explore whether MeVO could benefit from EVT. Hence, we conducted a multicentre study to compare the safety and efficacy of EVT for anterior circulation MeVO and LVO and to investigate the predictors of the good outcome at 90 days for MeVO patients.

## Methods

### Study population

Data from the Endovascular Treatment Key Technique and Emergency Workflow Improvement of Acute Ischemic Stroke (ANGEL-ACT) Registry database was used for current study. It was a nationwide prospective registry study that was conducted at 111 hospitals in 26 Chinese provinces between November 2017 and March 2019, enrolling 1793 consecutive adult LVO patients who underwent EVT. Jia *et al* 14 reported the full methods of ANGEL-ACT Registry earlier.

Exclusion criteria of the present study were: (1) patients who lack EVT records; (2) posterior circulation stroke; (3) tandem occlusion; and (4) missing values for the modified Rankin Scale (mRS).

### Data collection

The clinical and imaging data were prospectively collected in ANGEL-ACT Registry. All investigators recording the National Institutes of Health Stroke Scale (NIHSS) and mRS had been trained and certified. A neuroimaging core lab blinded to all clinical information evaluated all obtaining imaging. The assessment of the neuroimaging core lab included the occlusion site, Alberta Stroke Program Early CT Score (ASPECTS), tandem occlusion, LVO with underlying intracranial atherosclerosis disease (LVO-ICAD), which was defined as more than 70% residual stenotic degree or more than 50% residual stenotic degree with distal blood flow impairment or obvious target artery reocclusion tendency after mechanical thrombectomy (MT) during the procedure,^14^ angiographic recanalisation levels and haemorrhage transformation. For patients whose images were unavailable, we used site-reported data. Local researchers had identical training and experience with imaging interpretation with the neuroimaging core lab, with the exception that LVO-ICAD might potentially identify based on earlier imaging that showed a stenosis lesion at the occlusive location.

### Outcomes

**Figure 1:**
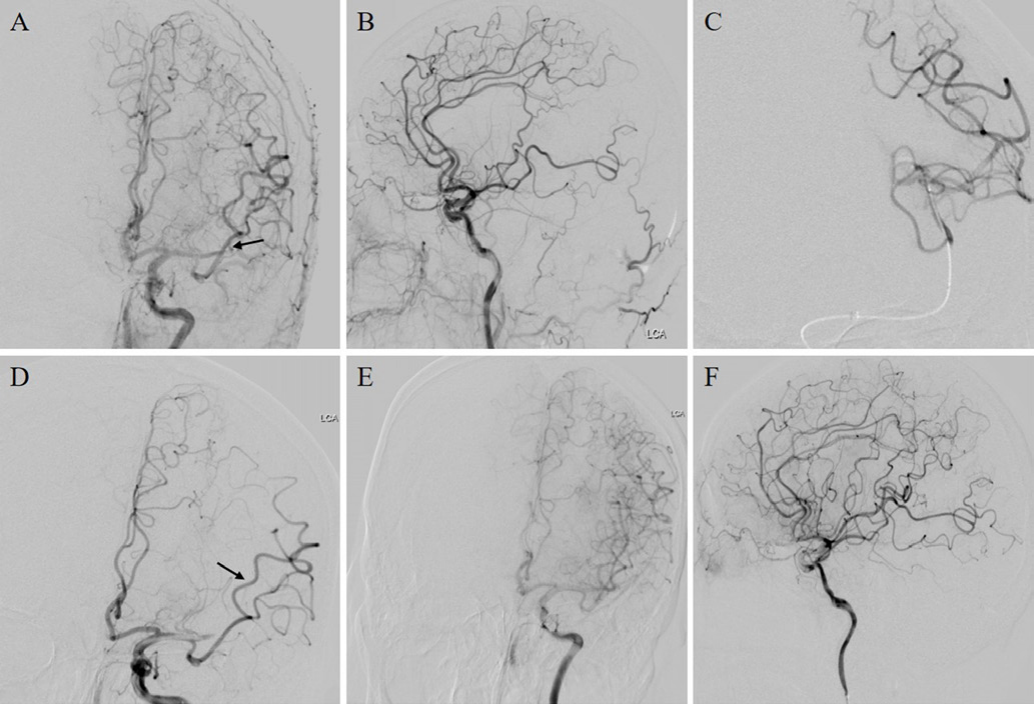
A MeVO recanalisation by the stent retriever. (A and B) Angiography showed an MCA-M2 branch occlusion (black arrow); (C) microcatheter angiography; (D) a stent retriever with 4–20 mm was deployed across the occlusion branch artery (black arrow); (E and F)recanalisation of the target branch artery after the first thrombectomy attempt. MCA, middle cerebral artery; MeVO, medium vessel occlusion.

In the present study, the mRS at 90 days was the primary outcome. mRS (0–1, 0–2 and 0–3) rates at 90 days and successful/complete recanalisation rates were the secondary outcomes. Mortality within 90 days, parenchymal haemorrhage (PH), intracranial haemorrhage (ICH) and symptomatic intracranial haemorrhage (SICH) within 24 hours after the procedure were the safety outcomes. We used mRS 0–2 and mRS 3–6 to categorise the 90-day good outcome and poor outcomes. We defined successful recanalisation as achieving the modified Thrombolysis in Cerebral Infarction (mTICI) level of no less than 2b at final angiography and complete recanalisation as achieving the mTICI level of 3 at final angiography (figure 1). We defined the first-pass recanalisation (FPR) as the successful recanalisation of the occlusion vessel with only one EVT device pass. Our study evaluated the SICH and PH based on Heidelberg Bleeding Classification.^14^

### MeVO identification

We identified MeVO according to the definition proposed by Goyal *et al*.^15^ MeVO in the anterior circulation should meet two criteria: (1) one of artery segment occlusions listed further: M2 segment (from the bifurcation/trifurcation of the main MCA to the circular sulcus of the insula), M3 segment (from the circular sulcus of the insula to the external/superior surface of the Sylvian fissure), A2 segment (from the origin of the anterior communicating artery to the origin of the callosomarginal artery), A3 segment (from the origin of the callosomarginal artery to the artery’s posterior turn above the corpus callosum) and (2) neurological deficit: a MeVO patient with the NIHSS ≥5 or NIHSS <5 with disabling deficit.^15^

### Statistical analysis

We conducted all the statistical analyses by SAS software V.9.4 (SAS Institute Inc). Baseline, procedural and outcome variables were displayed as median (IQR) or numbers (percentages) as appropriate in each group and compared using the Pearson χ^2^ test, Fisher’s exact or Mann-Whitney test. In order to compare the functional outcomes between the MeVO and LVO groups, we performed ordinal or binary logistic regression models to evaluate common odds ratio (OR) and ORs with their 95% confidence interval (CI), as appropriate. Then we divided MeVO population into good outcome and poor outcome groups and performed univariable analyses by Pearson χ^2^ test, Fisher’s exact or Mann-Whitney test. The variables with p<0.05 were the potential predictors. After that, our study used the variance inflation factor to evaluate multicollinearity. Next, the best cut-off values of baseline NLR, MT passes number and baseline NIHSS to predict the 90-day good outcome were calculated by receiver operating characteristic analyses. Then, with a removal criterion of p>0.05, we conducted a backward-stepwise logistic regression model to investigate the independent predictors of the 90-day good outcome. Statistically significant was determined at p<0.05 (two sided).

## Results

**Figure 2:**
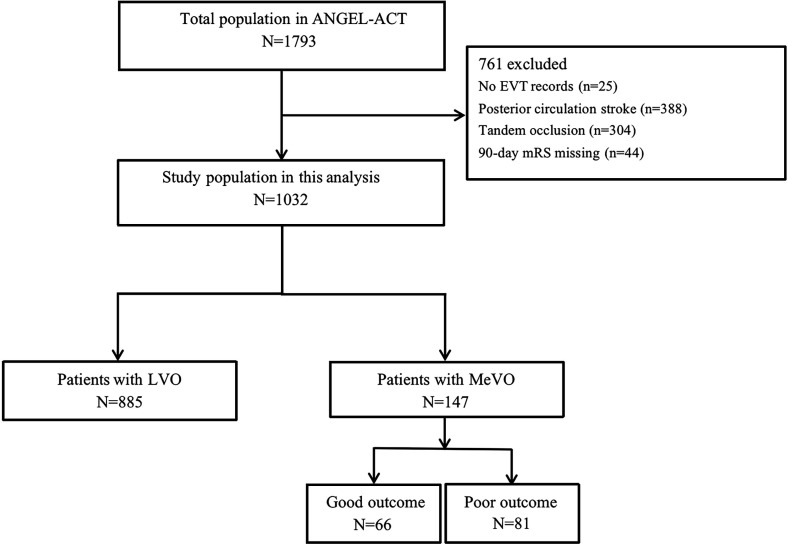
Flow chart of patient selection. EVT, endovascular treatment; LVO, large vessel occlusion; MeVO, medium vessel occlusion; mRS, modified Rankin Scale.

Figure 2 shows the flow chart of patient selection. Of the 1032 subjects included in the present study, 885 were LVO (intracranial internal carotid artery, MCA M1 segment or ACA A1 segment occlusions), and 147 were MeVO (MCA M2 segment or ACA A2 segment occlusions). Of the MeVO population, 66 achieved the 90-day good outcome and 81 achieved the 90-day poor outcome.

The comparison of baseline and procedural variables between MeVO and LVO groups is shown in table 1. MeVO patients had fewer passes (1 (1–2) vs 2 (1–3), p=0.001), higher baseline ASPECTS (10 (8–10) vs 9 (7–10), p=0.005) and shorter procedure duration (74 (45–105) min vs 80 (50–125) min, p=0.038) compared with LVO patients. MeVO patients less often received aspiration (10.2% vs 22.8%, p=0.001), balloon angioplasty (6.1% vs 15.1%, p=0.003), stenting (3.4% vs 12.2%, p=0.002) and glycoprotein (GP) IIb/IIIa receptor inhibitor (36.1% vs 48.8%, p=0.004) compared with LVO patients. On the other hand, the proportion of intra-arterial thrombolysis (IAT) (14.3% vs 6.2%, p=0.001) and FPR (57.5% vs 48.6%, p=0.047) were higher in the MeVO patients than LVO patients.

**Table 1:** Baseline and procedure characteristics of patients with MeVO and LVO

Baseline and procedure variables	All patients (n=1032)	MeVO (n=147)	LVO (n=885)	P value
Age, year, median (IQR）	66 (56–74)	67 (59–75)	66 (56–73)	0.120
Men, n (%)	620 (60.1)	75 (51.0)	545 (61.6)	**0.016**
Hypertension, n (%)	551 (53.4)	81 (55.1)	470 (53.1)	0.654
DM, n (%)	177 (17.2)	23 (15.7)	154 (17.4)	0.601
Hyperlipidaemia, n (%)	80 (7.8)	6 (4.1)	74 (8.4)	0.072
Coronary heart disease, n (%)	149 (14.4)	20 (13.6)	129 (14.6)	0.757
Atrial fibrillation, n (%)	408 (39.5)	59 (40.1)	349 (39.4)	0.872
Valvular heart disease, n(%)	82 (8.0)	6 (4.1)	76 (8.6)	0.061
Prior stroke, n (%)	222 (21.5)	36 (24.5)	186 (21.0)	0.343
Smoking history, n (%)				0.357
Never smoking	665 (64.4)	100 (68.0)	565 (63.8)	
Previous smoking	65(6.3)	11(7.5)	54(6.1)	
Current smoking	302(29.3)	36(24.5)	266(30.1)	
SBP, mm Hg	145 (130–160)	145 (132–160)	144 (130–160)	0.725
Baseline NIHSS*	15 (12–20)	16 (11–19)	15 (12–20)	0.673
ASPECTS†	9 (7–10)	10 (8–10)	9 (7–10)	**0.005**
Serum glucose, mmol/L, median (IQR)	6.8 (5.9–8.5)	6.7 (5.8–7.9)	6.8 (5.9–8.6)	0.440
Blood WBC, 10^9^/L, median (IQR)	8.2 (6.7–10.2)	8.1 (6.6–10.6)	8.3 (6.8–10.2)	0.816
NLR, median (IQR）	4.2 (2.5–6.9）	4.0 (2.5–7.0)	4.2 (2.5–6.8)	0.788
Pretreatment with antiplatelets, n (%)	163(15.8)	24(16.3)	139(15.7)	0.849
Pretreatment with IVT, n (%)	302 (29.3)	44 (29.9)	258 (29.2)	0.848
Underlying ICAD, n (%)				0.068
Yes	261 (25.3)	26 (17.7)	235 (26.6)	
No	670 (64.9)	566 (64.0)	104 (70.8)	
Undetermined	101 (9.8)	17 (11.6)	84 (9.5)	
General anaesthesia, n (%)	336 (32.6)	44 (29.9)	292 (33.0)	0.463
Stroke subtype by TOAST criteria				0.089
Large artery atherosclerosis	429 (41.6)	55 (37.4)	374 (42.3)	
Cardioembolism	447 (43.3)	62 (42.2)	385 (43.5)	
Other or unknown aetiology	108 (10.5)	24 (16.3)	84 (9.5)	
Undetermined	48 (4.7)	6 (4.1)	42 (4.8)	
Stent retriever, n (%)	940 (91.1)	133 (90.5)	807 (91.2)	0.780
Aspiration, n (%)	217 (21.0)	15 (10.2)	202 (22.8)	**0.001**
IAT, n (%)	76 (7.4)	21 (14.3)	55 (6.2)	**0.001**
Balloon angioplasty, n (%)	143 (13.9)	9 (6.1)	134 (15.1)	**0.003**
Stenting, n (%)	113 (11.0)	5 (3.4)	108 (12.2)	**0.002**
GP IIb/IIIa receptor inhibitor during EVT, n (%)	485 (47.0)	53 (36.1)	432 (48.8)	**0.004**
OTP, min, median (IQR)‡	300 (212–440)	286 (205–410)	300 (215–450)	0.168
Number of MT passes, median (IQR)	2 (1–3)	1 (1–2)	2 (1–3)	**0.001**
FPR, n (%)	514 (49.9)	84 (57.5)	430 (48.6)	**0.047**
Procedure duration, min, median (IQR)§	80 (50–120)	74 (45–105)	80 (50–125)	**0.038**
Intraprocedural embolisation, n (%)	59 (5.7)	6 (4.1)	53 (6.0)	0.356

Bold values indicate statistical significance.

*Four missing data.

†Five missing data.

‡ Thirteen missing data.

§ One missing data.

ASPECTS, Alberta Stroke Programme Early CT Score; DM, diabetes mellitus; EVT, endovascular treatment; FPR, first pass recanalisation; IAT, intra-arterial thrombolysis; ICAD, Intracranial atherosclerotic disease; IVT, intravenous thrombolysis; LVO, large vessel occlusion; MeVO, medium vessel occlusion; MT, mechanical thrombectomy; NIHSS, National Institute of Health Stroke Scale; NLR, neutrophil to lymphocyte ratio; OTP, Onset-to-puncture time; SBP, systolic blood pressure; TOAST, Trial of ORG 10172 in Acute Stroke Treatment; WBC, white cell count.

For the primary outcome, 90-day mRS distribution was similar in MeVO and LVO patients (3 (0–4) vs 3 (0–5), common OR = 1.00, 95% CI 0.73 to 1.38, P = 0.994). All secondary and safety outcomes between both groups were similar between MeVO and LVO groups (all p>0.05) (table 2).

**Table 2:** Outcome measures of patients with MeVO/LVO

Outcome variables	All patients (n=1032)	MeVO (n=147)	LVO (n=885)	(c)OR (95% CI)	P value	(c)OR (95% CI)	P value
Primary outcome							
90-day mRS, median (IQR)	3 (0–5)	3 (0–4)	3 (0–5)	1.07 (0.79 to 1.46)	0.657	1.00 (0.73 to 1.38)	0.994
Secondary outcomes							
90-day mRS 0–1, n (%)	422 (41.0)	63 (42.9)	359 (40.6)	1.10 (0.77 to 1.56)	0.601	0.98 (0.67 to 1.42)	0.909
90-day mRS 0–2, n (%)	464 (45.0)	66 (44.9)	398 (45.0)	1.00 (0.70 to 1.42)	0.987	0.90 (0.62 to 1.31)	0.589
90-day mRS 0–3, n (%)	583 (56.5)	87 (59.2)	496 (56.1)	1.14 (0.80 to 1.62)	0.477	1.06 (0.73 to 1.54)	0.764
Successful recanalisation, n (%)	926 (89.7)	132 (89.8)	794 (89.7)	1.01 (0.57 to 1.80)	0.977	1.00 (0.51 to 1.93)	0.992
Complete recanalisation, n (%)	737 (71.4)	108 (73.5)	629 (71.1)	1.13 (0.76 to 1.67)	0.552	1.08 (0.71 to 1.66)	0.710
Safety outcomes							
SICH within 24 hours, n (%)*	82 (8.3)	7 (4.8)	75 (8.9)	0.52 (0.23 to 1.14)	0.098	0.59 (0.26 to 1.34)	0.205
PH within 24 hours, n (%)†	104 (10.4)	12 (8.2)	92 (10.8)	0.74 (0.39 to 1.38)	0.344	0.84 (0.43 to 1.62)	0.596
Any ICH within 24 hours, n (%)†	249 (25.0)	34 (23.3)	215 (25.3)	0.90 (0.59 to 1.36)	0.610	1.04 (0.67 to 1.60)	0.877
Mortality within 90 days, n (%)	145 (14.1)	17 (11.6)	128 (14.5)	0.77 (0.45 to 1.33)	0.349	0.83 (0.48 to 1.46)	0.518

* Forty-two missing data

†Thirty-six missing data

(c)OR, (common) OR; ICH, intracranial haemorrhage; LVO, large vessel occlusion; MeVO, medium vessel occlusion; mRS, modified Rankin Scale; PH, parenchymal haemorrhage; SICH, symptomatic intracranial haemorrhage.

MeVO patients with good outcome had lower rate of atrial fibrillation (30.3% vs 48.2%, p=0.028), lower baseline NIHSS (13 (10–17) vs 17 (13–20), p=0.001), lower baseline NLR (3.4 (2.3–5.7) vs 4.9 (2.6–8.6), p=0.029), higher baseline ASPECTS (10 (9–10) vs 9 (7-10), p=0.016) and fewer MT passes (1 (1–2) vs 2 (1–2), p=0.003) compared with those presented with poor outcomes (online supplemental table). MeVO patients who achieved 90-day good outcome less often received stent retriever attempts (84.9% vs 95.1%, p=0.036) and more often underwent IAT attempts (21.2% vs 8.6%, p=0.030) than those with poor outcome. In the multivariate analysis (table 3), we observed baseline NLR≤4.1 (OR=2.13, 95% CI 1.14 to 3.99, p=0.019), MT passes ≤1 (OR=2.16, 95% CI 1.14 to 4.11, p=0.021) and baseline NIHSS ≤14 (OR=1.96, 95% CI 1.02 to 3.80, p=0.045) were associated with high chance of good outcome independently.

10.1136/svn-2022-001561.supp1 Supplementary data

**Table 3:** Independent predictors of 90-day good outome in patients with MeVO (back stepwise logistics, p<0.05)

Predictors	Or (95% CI)	P value
NLR ≤4.1 versus NLR >4.1	2.13 (1.14 to 3.99)	0.019
NIHSS ≤14 versus NIHSS >14	1.96 (1.02 to 3.80)	0.045
No. of MT passes ≤1 versus no. of MT passes >1	2.16 (1.14 to 4.11)	0.021

MeVO, medium vessel occlusion; MT, mechanical thrombectomy; NIHSS, National Institute of Health Stroke Scale; NLR, neutrophil-to-lymphocyte ratio.

## Discussion

In our study, 90-day mRS, successful recanalisation rate and SICH rate were similar between MeVO and LVO. Nevertheless, we found that the 90-day good outcome was positively related to baseline NLR≤4.1, baseline NIHSS ≤14 and the number of MT passes ≤1.

The safety and efficacy of EVT for MeVO have still remained unclear. Several factors may influence that, including the lack of routine utilisation of vascular imaging for patients with AIS before EVT or detecting MeVO could be a challenge.^15 16^ Additionally, the guideline or consensus on EVT for MeVO strokes still lacks.^17^ Because MeVO is noticeably heterogeneous in clinical presentation and can be highly disabling,^18^ medical management and intravenous thrombolysis (IVT) may be suboptimal for MeVO.^19 20^

In the present study, the successful recanalisation rate (89.8%) was comparable with a post hoc analysis that Coutinho *et al* 21 reported. However, our study had a lower 90-day good outcome rate (44.9%). This difference might be attributed to the higher NIHSS in the LVO group in their study, while in our study, the NIHSS was similar in MeVO and LVO groups. On the other hand, a meta-analysis suggested that despite similar recanalisation rates between M1 and M2 occlusion groups, the M2 occlusion group showed better good outcomes at 90 days and possibly lower mortality.^22^ The higher incidence of SICH in this group indicates an existing EVT-related risk for smaller and more fragile arteries, but this meta-analysis did not analyse the factors related to the better functional outcome for M2 occlusion patients.

Our neutral results were per those from what Coutinho *et al* 21 reported. Although the results showed similar recanalisation rates and functional outcomes between MeVO and LVO patients, we noticed several significant differences between patients with MeVO and those with LVO. MeVO patients had higher ASPECTS and cardioembolism stroke rates. The latest may explain the lower number of MT passes and higher FPR rate in the present study. Noteworthy, our further analysis showed that baseline NLR, NIHSS and the number of MT passes could predict a 90-day good outcome independently.

NIHSS has been widely used to assess the initial neurological deficits and the clinical stroke outcomes at 90 days.^23^ It was found to be associated with the clinical symptoms and the penumbra volume.^24^ Consistently, our results demonstrated a lower NIHSS in the good outcome group; this may be correlated with a higher ASPECTS, infrequent atrial fibrillation and lower NLR. Our study demonstrated that NIHSS ≤14 was noted to predict a 90-day good outcome independently. This result indicates that the NIHSS is an essential parameter related to EVT outcome in patients with MeVO.

Furthermore, this may also explain the lower number of MT passes in the good outcome group. A high NIHSS is generally associated with the larger ischaemic area, which mainly presents with a large thrombus,^25^ and it can be demonstrated reasonably that a more extensive thrombus is more challenging to remove. Another possibility is that the lower NIHSS may represent good collateral status, as better collateral circulation was associated with better clinical outcomes.^26^

As a marker of systemic inflammation, NLR could be an important marker for stroke prognosis.^27 28^ Moreover, higher NLR was independently associated with SICH in AIS patients undergoing IVT and EVT.^29^ The higher NLR in the poor outcome group may also align with the higher NIHSS in this group. The mechanism is that neutrophils could injure the blood–brain barrier and contribute to the injury of surrounding tissues, thus result in enlargement of the infarct area.^30–33^ Although not statistically significant, this finding was consistent with the evidence of higher SICH in the poor outcome group compared with the good outcome group (6.3% vs 3%) in the present study.

There is still controversy regarding the optimal number of MT passes for a good outcome in stroke patients. Linfante *et al* 34 reported that ≥3 passes could predict a poor outcome at 90 days in particular risk stratification. Bourcier *et al* 35 confirmed the latest finding though their study was limited to the sample size. Unlike them, we found the number of MT passes≤1 was one of the predictors of 90-day good outcome in MeVO patients. This might be explained by different study populations in the above three studies. Zaidat *et al* 36 reported that first-pass complete recanalisation could lead to better functional outcomes with decreased rates of death and procedural complications. Moreover, unlike the previous studies, which mainly concentrated on LVO, the smaller lumen and more fragile vessel wall in MeVO may increase the bleeding risk. In addition, the technical difficulties of MT may increase because of the smaller target artery in diameter and more distal clot location.^37^ Therefore, compared with LVO, the number of MT passes >1 indicates a greater risk of MeVO.

Our findings are in line with the ASTER trial,^38^ as it was confirmed that there should be no argument regarding the frontline MT strategy selection for MeVO. Although a different proportion of the EVT strategies were found between the good and poor outcome groups, the results of the multivariate analysis did not show any correlation between EVT strategies and the good outcome at 90 days. It was assumed that either aspiration or stent retriever technique has advantages and disadvantages; this might balance the outcome. A high recanalisation rate and a shorter procedure duration can be achieved by the aspiration technique.^37^ However, it is difficult to deliver the aspiration catheter to the occlusion site because of the larger size of the aspiration device,^39 40^ and the thrombus location and the arterial tortuosity might influence the success of the aspiration technique.^40^ Meanwhile, patients receiving the stent retriever might have faster recanalization, and those undergoing contact aspiration might have high recanalisation and low distal embolisation rates after first MT attempt.^40^

### Limitations

Several limitations should be acknowledged in the current study. First, although our multicentre study had a large sample size, the number of MeVO patients was still low. Thus, the statistical power may not be strong enough to reflect the actual influence of EVT on MeVO. Second, as the ANGEL-ACT registry did not enrol patients with MCA-M3/ACA-A3 segments occlusions, the majority of patients were M2 occlusion, and A2 occlusion only accounted for 8.8% of MeVO patients, which might overstate our findings. Third, variables such as proximal M2 occlusion and distal M2 occlusion were not collected, which might cause bias in our results. Finally, we used site-reported data because 33% of imaging was unavailable, which might introduce observation bias. Despite this, the bias was not evident since local researchers and the neuroimaging core lab all had identical training and experience with imaging interpretation.

## Conclusions

In this large multicentre registry study, 90-day mRS, successful recanalisation rate and SICH rate were similar between MeVO and LVO groups. However, patients with NIHSS ≤14, lower baseline NLR ≤4.1 and the number of MT passes ≤1 may gain more functional benefits from EVT. Further large RCTs are warranted.

## Data Availability

Data are available on reasonable request. Data are available upon reasonable request to the corresponding author.
