# Supplementary material for: Endovascular treatment for acute ischaemic stroke due to medium vessel occlusion: data from ANGEL-ACT registry
Source: Stroke Vasc Neurol. 2022 Sep 1;8(1):51–8. doi: 10.1136/svn-2022-001561 (PMC9985797; doi:10.1136/svn-2022-001561)
Supplement: Supplementary data [file svn-2022-001561supp001.pdf]

Supplementary Table. Baseline and procedure characteristics of MeVO patients with 90-day good outcome/poor outcome

Baseline and procedure variables	Good outcome (n=66)	Poor outcome (n=81)	P value
Age, y, median（IQR）	67(59-73)	67(60-76)	0.687
Men, n (%)	35(53.0)	40(49.4)	0.660
Hypertension, n (%)	32(48.5)	49(60.5)	0.145
DM, n (%)	10(15.2)	13(16.1)	0.882
Hyperlipidemia, n (%)	2(3.0)	4(4.9)	0.561
Coronary heart disease, n (%)	7(10.6)	13(16.1)	0.338
Atrial fibrillation, n (%)	20(30.3)	39(48.2)	**0.028**
Valvular heart disease, n (%)	2(3.0)	4(4.9)	0.561
Prior stroke, n (%)	13(20.0)	23(28.4)	0.223
Smoking history, n (%)			0.114
Never smoking	42(53.6)	58(71.6)	
Previous smoking	3(4.6)	8(9.9)	
Current smoking	21(31.8)	15(18.5)	
SBP, mmHg	145(132-160)	146(133-160)	0.833
Admission NIHSS	13(10-17)	17(13-20)	**0.001**
ASPECTS§	10(9-10)	9(7-10)	**0.016**
Serum glucose, mmol/L, median（IQR）	6.6(5.8-7.4)	7.0(5.8-8.6)	0.229
Blood WBC, 10^9/L, median（IQR）	7.6(6.8-8.8)	8.2(6.4-11.4)	0.241
NLR, median（IQR）	3.4(2.3-5.7)	4.9(2.6-8.6)	**0.029**
Pretreatment with antiplatelets, n (%)	8(12.1)	16(19.8)	0.213
Pretreatment with IVT, n (%)	46(69.7)	57(70.4)	0.929
Underlying ICAD, n (%)			
Yes	12(18.2)	14(17.3)	0.944
NO	47(71.2)	57(70.4)	
Undetermined	7(10.6)	10(12.4)	
General anesthesia, n (%)	14(21.2)	30(37.0)	**0.037**
Stroke subtype by TOAST criteria			0.498
Large artery atherosclerosis	27(40.9)	28(34.6)	
Cardioembolism	27(40.9)	35(43.2)	
Other or unknown etiology	11(16.7)	13(16.1)	
Undetermined	1(1.52)	5(6.2)	
Stent retriever, n (%)	56(84.9)	77(95.1)	**0.036**
Aspiration, n (%)	5(7.6)	10(12.4)	0.342
IAT, n (%)	14(21.2)	7(8.6)	**0.030**
Balloon angioplasty, n (%)	4(6.1)	5(6.2)	0.978

Stenting, n (%)	1(1.5)	4(4.9)	0.255
GP IIb/IIIa receptor inhibitor, n (%)	26(39.4)	27(33.3)	0.447
OTP, min, median（IQR）‖	265(210-405)	292(203-413)	0.791
Number of MT passes, median (IQR)	1(1-2)	2(1-2)	**0.003**
FPR, n (%)	42(64.6)	42(51.9)	0.121
Procedure duration, min, median（IQR）	70(39-97)	76(54-109)	0.100
Successful recanalization, n (%)	60(90.9)	72(88.9)	0.687
SICH, n (%)	2(3.0)	5(6.3)	0.605
Intraprocedural embolization, n (%)	2(3.0)	4(4.9)	0.871

Bold values indicate statistical significance.

§ 25 missing data; ‖ 5 missing data.

Abbreviations: IQR = interquartile range; DM = diabetes mellitus; WBC = white blood cell; NLR = neutrophil to lymphocyte ratio; IVT = intravenous thrombolysis; NIHSS = National Institute of Health Stroke Scale; ASPECTS = Alberta Stroke Program Early CT Score; ICAD = Intracranial atherosclerotic disease; IAT = intra-arterial thrombolysis; MT = mechanical thrombectomy; FPR = first pass recanalization; OTP = Onset-to-puncture time; SBP = systolic blood pressure; TOAST = Trial of ORG 10172 in Acute Stroke Treatment.
